# A Structured Curriculum for Interprofessional Training of Emergency Medicine Interns

**DOI:** 10.5811/westjem.2019.11.44139

**Published:** 2019-12-18

**Authors:** Ashley C. Rider, Tiffany C. Anaebere, Mariko Nomura, David Duong, Charlotte P. Wills

**Affiliations:** * Highland Hospital, Alameda Health System, Department of Emergency Medicine, Oakland, California; † Dignity Health, St. Joseph’s Medical Center, Department of Emergency Medicine, Stockton, California

## Abstract

Interprofessional education (IPE) has been shown to improve health outcomes and patient satisfaction. IPE is now represented in the Accreditation Council for Graduate Medical Education’s emergency medicine (EM) milestones given the team-based nature of EM. The Highland Allied Health Rotation Program (H-AHRP) was developed by residents to enhance and standardize IPE for EM residents in a single hospital setting. H-AHRP was incorporated into the orientation month for interns starting in the summer of 2016. EM interns were paired with emergency department preceptors in registered nursing (RN), respiratory therapy (RT), pharmacy (PH), laboratory (LAB), and social work (SW) in either a four-hour shadowing experience (RN, RT, PH) or lecture-based overview (LAB, SW). We conducted a survey before and after the program. Overall, the EM interns reported an improved understanding of the scope of practice and day-to-day logistics after working with the preceptors. They found the program helpful to their future as physicians and would recommend it to other residencies. The H-AHRP program allows for the early incorporation of IPE into EM training, enhances interns’ understanding of both the scope and logistics of their colleagues, and is a well-received effort at improving team-based care.

## BACKGROUND

Almost 20 years ago, the Institute of Medicine (now the National Academy of Medicine) issued a report drawing attention to the high rate of preventable healthcare errors, many of which may have been influenced by ineffective teamwork.^1^ The increased awareness prompted numerous studies demonstrating how interprofessional teams can positively impact patient satisfaction, acceptance of care, and improve health outcomes.^2^

Interprofessional education (IPE) is now represented in the competencies for emergency medicine (EM) training.^3^ The Accreditation Council for Graduate Medical Education and the American Board of Emergency Medicine developed the EM milestones, which include skills such as effective communication and teamwork, yet formal interprofessional education is often lacking. The Highland Allied Health Rotation Program (H-AHRP) was developed to provide deliberate exposure to the role of nursing, pharmacy, respiratory therapy, social work, and laboratory services during the first month of residency.

## OBJECTIVES

The program’s objectives for first-year residents were threefold: 1) better understand the roles of their fellow health professionals (scope); 2) learn to perform a number of procedures and actions common to these roles (logistics); and 3) develop skills of interprofessional communication and teamwork while getting to know these team members. The desired outcome was a resident physician who understands the contributions of other healthcare professionals, integrates skill sets effectively, and champions an interdisciplinary approach to patient care.

## CURRICULAR DESIGN

At Highland Hospital the first month of intern year serves as an orientation to the emergency department (ED) during which interns participate in ED shifts, lectures, and workshops. The H-AHRP program was initially created in 2016 to purposefully introduce IPE into the curriculum. After significant modifications and a pilot year, the program was studied in 2018.

At the beginning of orientation month, an introductory presentation and syllabus were provided to outline the expectations and objectives for the program. EM interns were assigned sessions with registered nursing (RN), respiratory therapy (RT), pharmacy (PH), laboratory (LAB), and social work (SW). The RN, RT, and PH shifts were one-on-one sessions lasting four hours, during which the intern participated in the activities of his or her preceptor with the guidance of syllabus objectives. These sessions allowed the interns to experience the real-time responsibilities of each allied health professional. In addition, SW and LAB learning objectives were introduced through a group-based tour and discussion, with the respective experts, the ED medical social worker and the director of the clinical laboratory, guiding the session.

To evaluate the interns’ understanding, we administered pre- and post-program surveys using a five-point modified Likert scale with responses from −2 (strongly disagree) to +2 (strongly agree). The numerical responses were averaged for each question and these values were aggregated based on the professional it referenced and question type. The five sections that followed asked questions about the role of each professional represented in the program. We subdivided these questions into “scope”-type questions or “logistic”-type questions. “Scope” referred to questions related to the intern’s understanding of the general role or scope of practice of that profession, whereas “logistic” referred to specific procedures or actions of that profession. In the post-program survey, there were 10 additional questions aimed at collecting general program feedback and perceived utility of the session.

## IMPACT/EFFECTIVENESS

H-AHRP was designed and implemented to fill a need for improved IPE early on in EM training. After participating in this program, interns showed an overall trend toward increased understanding of the scope of practice and logistics for each professional group. During the initial orientation lecture, all 12 interns (100%) completed the pre-program survey, and 11 of 12 (92%) completed the similar post-program survey at the end of the month. All responses to the seven general interprofessional questions demonstrated a better appreciation of IPE after the intervention, from agree (+1.0) to closer to strongly agree (+1.7).

Overall, interns reported an improved understanding of both scope and logistics of each profession after the program (Figure). For example, on the pre-program survey interns reported the least understanding of the scope of practice of respiratory therapists compared to other professions and largely disagreed with statements of understanding. After the session with the RTs, interns went from disagree (−0.6) to closer to strongly agree (+1.6) with statements of understanding of scope and a similar two-point jump from disagree (−1.0) to agree (+1.0) for logistics of RTs. We saw positive trends across all specialties, particularly for questions related to logistics. In the post-program survey, a 10-question section was included for general feedback. Overall, participants agreed on the program’s helpfulness to their future and would recommend a similar program to other EM residencies.

Although limited in time and scope, as well as by its small studied sample size, this IPE initiative serves as a framework for EM residencies to introduce the basic roles and skills of non-physician team members in the ED. We recognize the limitations of survey data and self-report; therefore, future studies should aim to objectively evaluate the impact of IPE on physician behavior and patient care over time.

Excellent teamwork is predicated on an understanding of the skills and knowledge of teammates. As Wilbur describes in a 2014 call to action, EM is the best-qualified specialty to lead an emphasis on IPE.^4^ The H-AHRP is an example of a structured curriculum with clear objectives for EM interns to learn the basic scope and logistical roles of emergency nurses, respiratory therapists, pharmacists, laboratory scientists, and social workers to provide a foundation for IPE. By formally integrating H-AHRP into intern year, we hope to promote ED collaboration for effective, team-based patient care in residency and beyond.

## Supplementary Information



## Figures and Tables

**Figure:**
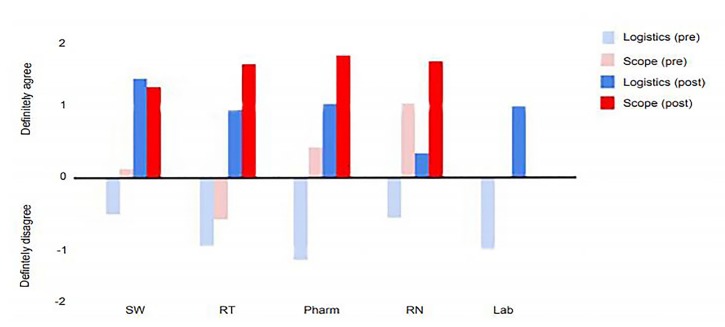
Survey results by specialty from the pre-and post-program survey taken by interns after they participated in an allied health professions program designed to enhance their understanding of the work done by non-physician team members in the emergency department. *SW*, social work; *RT*, respiratory therapy; *Pharm*, pharmacy; *RN*, registered nursing; *Lab*, laboratory
