# Supplementary material for: A Structured Curriculum for Interprofessional Training of Emergency Medicine Interns
Source: West J Emerg Med. 2019 Dec 18;21(1):149–51. doi: 10.5811/westjem.2019.11.44139 (PMC6948681; doi:10.5811/westjem.2019.11.44139)
Supplement: Supplementary file 1 [file wjem-21-149-s001.docx]

Appendix A: Procedures and Objectives for each H-AHRP session


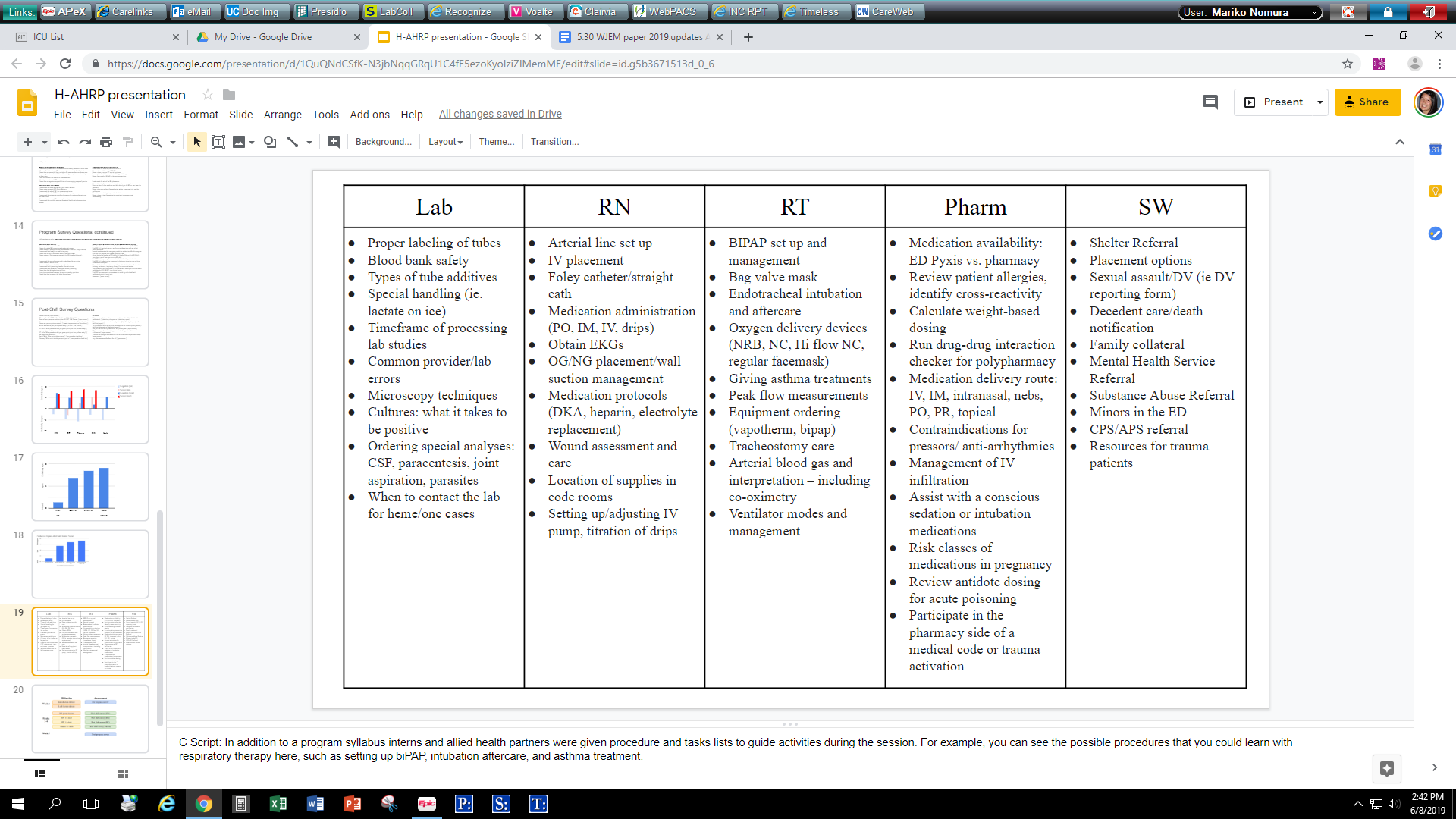


Appendix B: Pre- and Post-Program Survey

**Pre- and Post H-AHRP Program Survey**

All questions on scale: Definitely Disagree (-2), Slightly Disagree (-1), Neutral (0), Slightly Agree (+1), Definitely Agree (+2), n/a

**General Interprofessional Teamwork**

I am able to promote effective communication among members of an IP team

I feel comfortable using an IP team approach to assess the health situation

I know how to learn with, from, and about IP team members to enhance care

I can recognize how others' skills and knowledge complement and overlap with my own

I take into account the ideas of IP team members

I develop care plans with IP team members

I know how to negotiate responsibilities with overlapping scopes of practice

***Emergency Dept Social Work***

I understand the scope of practice of ED Social Workers

I know when to consult ED Social Workers

I understand the role of SW in a trauma resuscitation

I understand the role of SW in a domestic violence report

I understand the options for possible placements for patients who can't care for themselves

I know where to access SW resources for patients

I understand the available resources for mental health and substance abuse referral

***Emergency Dept Respiratory Therapy***

I understand the scope of practice of ED RTs

I know when and how to call ED RTs

I know where to access RT special equipment

I can explain the different methods of oxygen delivery

I know how to adjust BiPAP or the ventilator settings

***Emergency Dept Pharmacy***

I understand the role of the ED pharmacist

I know the role of pharmacy in med codes and trauma resuscitations

I have an idea of what meds are available directly in the ED vs. sent from the pharmacy

I know resources to check for medication advice, cross-reactivity, and the antibiogram

I know common dosing for procedural sedation

I know where to look for medication questions in pregnancy and breastfeeding

***Emergency Dept Nursing***

I understand the scope of the ED nurse

I know the role of ED nurses in med codes and trauma

I know where to get supplies and place common items (IV, foley, NG tube)

I understand how to adjust a medication pump

I know how to apply the cardiac monitor and EKG leads

I know where to find common protocols (ie DKA, etoh withdrawal)

***Laboratory***

I understand the steps of how to safely order blood for my patient

I know where to locate the lab

I understand the types of additives in each tube

I understand basic processing time for labs and cultures

I understand how to properly label tubes for lab processing

I know how the lab handles special fluids

I can give examples of common lab errors caused by providers

I know how best to contact the lab with questions

**General Program (only included on Post H-AHRP Program Survey)**

I benefited from the exposure to allied health services through H-AHRP

H-AHRP will positively impact my future collaborations with my allied health colleagues

I would recommend other emergency medicine residencies offer this program

I felt that this program was a good use of my time

Given the limited number of ED shifts in 4 weeks, I felt an H-AHRP shift was a better use of time than an extra ED shift

H-AHRP will impact my future work with allied health professionals

H-AHRP will make it easier to engage in challenges in patient care with my allied health colleagues

My medical school exposed me to working with allied health professionals (nursing, social work, respiratory therapy) in a structured manner

After graduating medical school, I felt comfortable working with allied health professionals (RN/SW/RT) in a clinical setting.

H-AHRP has competently prepared me for working with allied health professionals during intern year

Comments: [ open answer]

Appendix C: Post-Shift Survey

**Post-Shift H-AHRP Survey**

Date of rotation [ open answer ]

What number H-AHRP shift is this for you? [ 1, 2, 3, 4, 5 ]

Name of your preceptor and discipline (RN, RT, SW, Pharm): [ open answer ]

Please rate your preceptor today: [1 (needs improvement) to 5 (excellent) ]

Please rate your experience overall: [1 (needs improvement) to 5 (excellent) ]

Which rotation did you participate in today? [ RN, RT, SW, Pharm ]

RN shifts: What procedures did you get to participate in or perform today? [ see procedure checklists ]

RT shifts: What procedures did you get to participate in or perform today? [ see procedure checklists ]

Social Work: What topics did you cover? [ see procedure checklists ]

Pharmacy: What activities did you participate in? [ see procedure checklists ]

All shifts:

This experience helps me better understand the work of this allied health professional: [1 (definitely disagree) to 5 (definitely agree) ]

This experience makes me a better physician: [1 (definitely disagree) to 5 (definitely agree) ]

This experience helps me to better collaborate on an interdisciplinary team: [1 (definitely disagree) to 5 (definitely agree) ]

Did you find this activity beneficial? Why or why not? [ open answer ]

What are the weaknesses or things you would change about this shift/rotation? [ open answer ]

What are the strengths or elements of this shift/rotation that you would keep? [ open answer ]

Any other comments/feedback for us? [ open answer ]
